# Hydralazine protects the heart against acute ischaemia/reperfusion injury by inhibiting Drp1-mediated mitochondrial fission

**DOI:** 10.1093/cvr/cvaa343

**Published:** 2021-01-02

**Authors:** Siavash Beikoghli Kalkhoran, Janos Kriston-Vizi, Sauri Hernandez-Resendiz, Gustavo E Crespo-Avilan, Ayeshah A Rosdah, Jarmon G Lees, Joana Rodrigues Simoes Da Costa, Naomi X Y Ling, Jessica K Holien, Parisa Samangouei, Kroekkiat Chinda, En Ping Yap, Jaime A Riquelme, Robin Ketteler, Derek M Yellon, Shiang Y Lim, Derek J Hausenloy

**Affiliations:** 1 The Hatter Cardiovascular Institute, Institute of Cardiovascular Science, University College, 67 Chenies Mews, WC1E 6HX London, UK; 2 Cardiovascular and Metabolic Disorder Programme, Duke-NUS Medical School, 8 College Road, 169857, Singapore; 3 National Heart Research Institute Singapore, National Heart Centre, 5 Hospital Drive, 169609, Singapore; 4 MRC Laboratory for Molecular Cell Biology, University College, Gower St, Kings Cross, WC1E 6BT London, UK; 5 Department of Biochemistry, Medical Faculty, Justus Liebig-University, Ludwigstraße 23, 35390 Giessen, Germany; 6 O’Brien Institute Department, St Vincent’s Institute of Medical Research, 9 Princes Street Fitzroy Victoria, 3065, Australia; 7 Faculty of Medicine, Universitas Sriwijaya, Palembang, Bukit Lama, Kec. Ilir Bar. I, Kota Palembang, 30139 Sumatera Selatan, Indonesia; 8 Department of Surgery and Medicine, University of Melbourne, Medical Building, Cnr Grattan Street & Royal Parade, 3010 Victoria, Australia; 9 Metabolic Signalling Laboratory, St Vincent’s Institute of Medical Research, School of Medicine, University of Melbourne, Melbourne, Victoria, Australia; 10 St Vincent’s Institute of Medical Research, 9 Princes Street, Fitzroy Victoria, 3065, Australia; 11 ACRF Rational Drug Discovery Centre, St Vincent’s Institute of Medical Research, 9 Princes Street Fitzroy Victoria, 3065, Australia; 12 Department of Physiology, Faculty of Medical Science, Naresuan University, Tha Pho, Mueang Phitsanulok, 65000, Thailand; 13 Advanced Center for Chronic Disease (ACCDiS), Facultad de Ciencias Químicas y Farmacéuticas & Facultad de Medicina, Universidad de Chile, Sergio Livingstone 1007, Independencia, Santiago, Chile; 14 Yong Loo Lin School of Medicine, National University Singapore, 1E Kent Ridge Road, 119228, Singapore; 15 Cardiovascular Research Center, College of Medical and Health Sciences, Asia University, Lioufeng Rd., Wufeng, 41354 Taichung, Taiwan

**Keywords:** Hydralazine, Cardioprotection, Acute myocardial ischaemia/reperfusion injury, Mitochondrial fission

## Abstract

**Aims:**

Genetic and pharmacological inhibition of mitochondrial fission induced by acute myocardial ischaemia/reperfusion injury (IRI) has been shown to reduce myocardial infarct size. The clinically used anti-hypertensive and heart failure medication, hydralazine, is known to have anti-oxidant and anti-apoptotic effects. Here, we investigated whether hydralazine confers acute cardioprotection by inhibiting Drp1-mediated mitochondrial fission.

**Methods and results:**

Pre-treatment with hydralazine was shown to inhibit both mitochondrial fission and mitochondrial membrane depolarisation induced by oxidative stress in HeLa cells. In mouse embryonic fibroblasts (MEFs), pre-treatment with hydralazine attenuated mitochondrial fission and cell death induced by oxidative stress, but this effect was absent in MEFs deficient in the mitochondrial fission protein, Drp1. Molecular docking and surface plasmon resonance studies demonstrated binding of hydralazine to the GTPase domain of the mitochondrial fission protein, Drp1 (KD 8.6±1.0 µM), and inhibition of Drp1 GTPase activity in a dose-dependent manner. In isolated adult murine cardiomyocytes subjected to simulated IRI, hydralazine inhibited mitochondrial fission, preserved mitochondrial fusion events, and reduced cardiomyocyte death (hydralazine 24.7±2.5% vs. control 34.1±1.5%, *P*=0.0012). In *ex vivo* perfused murine hearts subjected to acute IRI, pre-treatment with hydralazine reduced myocardial infarct size (as % left ventricle: hydralazine 29.6±6.5% vs. vehicle control 54.1±4.9%, *P*=0.0083), and in the murine heart subjected to *in vivo* IRI, the administration of hydralazine at reperfusion, decreased myocardial infarct size (as % area-at-risk: hydralazine 28.9±3.0% vs. vehicle control 58.2±3.8%, *P*<0.001).

**Conclusion:**

We show that, in addition to its antioxidant and anti-apoptotic effects, hydralazine, confers acute cardioprotection by inhibiting IRI-induced mitochondrial fission, raising the possibility of repurposing hydralazine as a novel cardioprotective therapy for improving post-infarction outcomes.

## 1. Introduction

Although, mortality following acute myocardial infarction (AMI) is on the decline, the prevalence and severity of post-AMI heart failure (HF) is on the rise. Therefore, new treatments are needed to protect the myocardium against the detrimental effects of acute ischaemia/reperfusion injury (IRI), in order to reduce myocardial infarct (MI) size, preserve cardiac function, and prevent the onset of HF.^1–3^ The maintenance of normal mitochondrial respiratory function is of critical importance to the heart, given the high energy demands required for contractile function. Under conditions of energetic stress, such as experienced during acute myocardial IRI, mitochondrial dysfunction is a key determinant of cardiomyocyte death and cardiac dysfunction. As such, new therapies capable of preventing mitochondrial dysfunction during acute myocardial IRI, may provide novel strategies for cardioprotection.^4^^,^^5^

In response to acute myocardial IRI, mitochondria undergo fission resulting in mitochondrial dysfunction and cardiomyocyte death,^6^^,^^7^ a process which is regulated by the mitochondrial fission protein, dynamin-related peptide-1 (Drp1),^8^ whose mitochondrial fission properties is dependent on its GTPase activity.^9^ Genetic^10^^,^^11^ and pharmacological inhibition of Drp1 using the putative Drp1 inhibitor, Mdivi-1,^12^ have been shown to attenuate cell death in isolated cardiomyocytes, and reduce MI size in the rodent heart subjected to acute IRI,^7^^,^^13^ demonstrating IRI-induced mitochondrial fission to be an important target for cardioprotection. However, recent studies have reported Mdivi-1 to display off-target mitochondrial effects that are independent of its inhibitory effects on Drp1 GTPase activity.^14^^,^^15^ As such, new treatments are needed to inhibit Drp1-mediated mitochondrial fission, in order to translate this cardioprotective strategy for patient benefit.

Experimental animal studies have reported that hydralazine, a pharmacological agent that is used to treat patients with hypertension and chronic HF,^16^^,^^17^ can protect the heart against acute IRI, but the mechanisms underlying this cardioprotective effect remain unclear.^18^^,^^19^ In this study, we report that the acute administration of hydralazine protects the heart against the detrimental effects of acute IRI by inhibiting Drp1-mediated mitochondrial fission.

## 2. Methods

All animal procedures conformed to the guidelines from Directive 2010/63/EU of the European Parliament on the protection of animals used for scientific purposes. Animal experiments were conducted in accordance with the Animals (Scientific Procedures) Act 1986 published by the UK Home Office and the Guide for the Care and Use of Laboratory Animals published by the US National Institutes of Health 1996, and in compliance with the Singapore National Advisory Committee for Laboratory Animal Research guidelines. Mitochondrial dynamics were assessed in the adult heart using female Dendra2 mice {Jackson-[B6; 129S-Gt(ROSA)26Sortm1(CAG-COX8A/Dendra2) Dcc/J]}, which have been engineered to express the mitochondrial protein, Dendra2, a photo-switchable (green to red), monomeric fluorescent protein derived from octocoral *Dendronephthya sp*.^20^ Isolated perfused *ex vivo* heart IRI studies were performed using female C57/BL6 mice (InVivos Jackson Laboratory), and heart *in vivo* IRI studies were performed using male C57Bl/6N mice (InVivos Jackson Laboratory). Unless otherwise stated, all laboratory reagents were purchased from Sigma.

### 2.1 Molecular docking studies

Molecular docking studies were performed to investigate whether hydralazine can bind to the GTPase domain of Drp1 and OPA1. Crystolagraphic waters and the ligand (phosphoaminophosphonic acid-guanylate ester) were removed from the X-ray crystal structure of the GTPase domain of Drp1 (PDB code: 1H1V^21^) and hydrogen atoms were added. A homology model of OPA1 (obtained from swissmodel.expasy.org) was used. A single low energy 3D structure of hydralazine was created using MarvinSketch (http://www.chemxon.com). This was then docked into the prepared GTPase domain of Drp1 or OPA1 using Surflex-Dock (Sybylx2.1.1, Certara L.P). The docking protocol was generated using default parameters, with a threshold of 0.5 and no bloat. The Suflex-Dock GeomX docking module was utilized with full flexibility to allow for ligands, ligand rings, protein hydrogen atoms, and protein heavy atoms. Furthermore, six additional starting conformations of hydralazine were requested. All other parameters were kept as default. The top 20 docked solutions (based on scoring function) for hydralazine were retained and visually analysed, and the major cluster was selected as the proposed binding mode.

### 2.2 Surface plasmon resonance binding assay

In order to determine whether hydralazine can bind to Drp1, we performed a surface plasmon resonance (SPR) binding assay using a Biacore T200 (GE), and analysed the data using the BIAevaluation software package. His-tagged recombinant Drp1 protein was diluted 1:100 in immobilization buffer containing HEPES 10 mM, NaCl 0.15 M, EDTA 50 µM, Tween 20 0.005%, pH 7.5, and then tethered to a XanTec NiHC 1200 chip (XanTec Bioanalytics) as per manufacturer’s instructions. The final amount on the surface was 10 000 RU. Flow cell 1 was derivatised and blocked for use as a reference. An unrelated protein (PA2G4) was tethered to Flow cell 3 and used as a reference. Hydralazine hydrochloride was diluted in immobilization buffer in a two-fold concentration series from 500 to 0.1 µM, and injected across the chip for 60 s at 30 µL/min. Experiments were conducted in duplicate in three independent experiments.

### 2.3 Drp1 GTPase activity

We investigated whether hydralazine has the ability to inhibit the GTPase activity of Drp1, which is required for the latter to mediate mitochondrial fission.^9^ For bacterial expression of recombinant human Drp1 (N-term his6) protein, cDNA for human Drp1 (Isoform2, Uniprot ID: O00429-3, kindly provided by Prof Michael Ryan, Monash University, Australia) was cloned into pQE-30 vector and transformed in Rosetta (DE3) competent cells (Novagen, Merck Millipore). Transformed cells were propagated in 2L of Luria–Bertani broth, in the presence of 100 µg/mL ampicillin at 37°C, 120 rpm to *A*_600_=2.5. Protein expression was initiated by the addition of 0.5 mM IPTG, after which, cultures were incubated at 16°C for 18 h. Cells were harvested by centrifugation at 3500 rpm (20 min), and the pellet was re-suspended in lysis buffer containing Tris–HCl (50 mM, pH 7.3), NaCl (0.5 M), imidazole (50 mM), glycerol (5%), β-mercaptoethanol (2 mM), LEUPEP (0.1 mM), AEBSF (0.1 mM) and benzaminidium chloride (1 mM). The cells were lysed using a precooled EmulsiFlex-C5 homogenizer (Avestin) on ice and clarified by centrifugation at 20 000 rpm for 30 min. The clarified cell lysate was loaded onto a 5 mL Nickel Chelating Sepharose Fast Flow column (GE Healthcare, Buckinghamshire, UK). The column was rinsed with ‘wash buffer’ containing Tris–HCl (50 mM, pH 7.6), NaCl (150 mM), glycerol (10%) and β-mercaptoethanol (2 mM), and Drp1 protein was eluted with wash buffer supplemented with 400 mM imidazole. Eluted proteins were equilibrated with a buffer containing Tris–HCl (50 mM, pH 7.6), NaCl (150 mM), glycerol (10%) and TCEP (2 mM) using a PD-10 desalting column (GE Healthcare). Recombinant human Drp1 (100 ng) was incubated with vehicle control or hydralazine hydrochloride for 30 min at 37°C. GTPase activity of Drp1 was determined using a GTPase assay kit (Novus Biologicals, CO, USA) according to the manufacturer’s instructions.^22^

### 2.4 Cell studies investigating H_2_O_2_-induced mitochondrial effects

HeLa cells (ATCC-CCL2) cultured in DMEM-Glutamax (ThermoFisher) were transfected with mitochondrial-targeted red fluorescent protein (RFP) plasmid (1:3 ratio) using Xtreme-gene-9 transfection agent (Roche) and incubated for 48 h before experiments. For mitochondrial membrane potential studies, HeLa cells were incubated with rhodamine 123 (2 μM) for 30 min at 37°C.

To investigate whether hydralazine can inhibit mitochondrial fission and prevent mitochondrial membrane depolarization induced by oxidative stress, cells were treated with either hydralazine (1 µM) (*N*=6 independent experiments) or distilled water vehicle control (*N*=5 independent experiments) for 40 min at 37°C and then subjected to H_2_O_2_ (3.3 mM) for 60 min.^23^ A separate group (*N*=3 independent experiments) of baseline cells did not receive either vehicle control, hydralazine, or H_2_O_2_. The cells were imaged using a Leica xSP5 confocal microscope (×40 magnification) in Tyrode’s buffer: NaCl (137 mM), KCl (5 mM), MgCl_2_ (0.4 mM), CaCl_2_ (1 mM), D-Glucose (10 mM), and Na HEPES (10 mM) at pH 7.4. For the baseline cells, six randomly acquired confocal images containing ∼14 cells per image were assessed in each independent experiment. For the H_2_O_2_ studies, eight randomly acquired confocal images containing ∼9 cells per image were assessed in each independent experiment. For mitochondrial morphology, cells were allocated to show either mainly (>50%) mitochondrial fragmentation or mitochondrial elongation. For the mitochondrial membrane potential studies, rhodamine 123 fluorescence was analysed using the Otsu thresholding algorithm in ImageJ.^24^

We next evaluated the mitochondrial effects of hydralazine in Drp1 wild***-***type (WT) and Drp1 KO mouse embryonic fibroblasts (MEFs) subjected to H_2_O_2_. The Drp1 WT and KO MEFs were kind gifts from Dr Hiromi Sesaki (Johns Hopkins University School of Medicine, USA), and were isolated from embryos of WT and transgenic C57BL/6 mice as described in the References.^25^^,^^26^ To generate the mitochondria-reporter cell lines, Drp1 WT and KO MEFs were transduced with pAS2.EYFP.puro lentiviruses (RNAiCore, Academia Sinica, Taiwan) carrying the mitochondrial-targeted GFP sequence. Transduced cells were purified by fluorescence-activated cell sorting and propagated in growth media containing DMEM high glucose (Lonza) supplemented with 10% non-heat inactivated foetal calf serum (Sigma-Aldrich), 1% L-alanyl-L-glutamine dipeptide (GlutaMAX; Invitrogen), 100 μg/mL streptomycin and 0.25 μg/mL amphotericin B (Lonza).

To simulate oxidative stress-induced cell injury, cells were seeded on 0.1% gelatin-coated plates at 10 526 cells/cm^2^ overnight and subjected to H_2_O_2_ treatment. Cells were treated with either 1 µM hydralazine or distilled water vehicle control for 40 min at 37°C and then subjected to 3.3 mM H_2_O_2_ for 120 min in the presence of hydralazine or distilled water vehicle control. Cell death was assessed with propidium iodide (3 µg/mL, Sigma-Aldrich, Thermo Fisher Scientific) and Hoechst 33258 (3 µg/mL, Sigma-Aldrich). The number of dead cells (as indicated by propidium iodide staining) were counted and expressed as a percentage over the total number of cells (Hoechst 33258 positive). At least 1200 cells were counted per group for each of the nine independent experiments.^27^

For the mitochondrial morphology studies, mitochondrial-reporter MEFs were fixed with 4% paraformaldehyde. Images were captured at 600× magnification with a fluorescence microscope (Olympus BX60) and analysed with ImageJ software. For mitochondrial morphology, cells were allocated to show either mainly (>50%) mitochondrial fragmentation or mitochondrial elongation. At least 60 cells were counted per group for each of the 11 independent experiments. The proportion of each category was expressed as percentage relative to the total number of cells per group.^27^^,^^28^

Mitochondrial membrane potential was assessed using tetra-methyl rhodamine methyl ester (TMRM), a lipophilic cation that accumulates selectively into the mitochondria according to the mitochondrial membrane potential. Cells were incubated with a non-quenching dose of TMRM at 10 nM in culture media. Carbonyl cyanide 3-chlorophenylhydrazone (CCCP, 50 µM), a mitochondrial respiratory uncoupler, was used as a positive control to induce dissipation of mitochondrial membrane potential. Images were captured at 200× magnification with a fluorescence microscope (Olympus IX71) and fluorescence intensity was assessed semi-quantitatively using ImageJ software. At least 450 cells from 3 random fields were counted per group for each of the 7 independent experiments.

Mitochondrial production of reactive oxygen species (ROS) was assessed using MitoSOX Red (Thermo Fisher Scientific). Cells treated with 5 mM of the antioxidant N-acetyl-L-cysteine (Sigma-Aldrich) were used as a positive control. Images were captured at 200× magnification with a fluorescence microscope (Olympus IX71) and fluorescence intensity was assessed semi-quantitatively using ImageJ software. At least 450 cells from 3 random fields were counted per group for each of the 7 independent experiments.^27^^,^^28^

### 2.5 Adult murine ventricular cardiomyocyte simulated IRI studies

We investigated whether hydralazine has the ability to reduce cell death, inhibit mitochondrial fission, and promote mitochondrial fusion events in adult murine ventricular cardiomyocytes following simulated ischaemia/reperfusion injury (SIRI). Murine ventricular cardiomyocytes were isolated from adult Dendra2 mice using liberase digestion.^29^ Mice were anaesthetized via intraperitoneal injection of ketamine (65 mg/kg) and xylazine (13 mg/kg). Anticoagulant heparin sodium (Rockhardt UK Ltd, Wrexham, UK) at a dose of up to 5000 units/kg body weight was co-administered with the anaesthetic. Upon the onset of deep anaesthesia, identified as the loss of the pedal pain withdrawal reflex, slowing of heart rate and breathing, the mice were euthanized by excising the heart. Harvested adult murine hearts were Langendorff-perfused using ‘isolation buffer’ containing NaCl (113 mM), KCl (4.7 mM), KH_2_PO_4_ (0.6 mM), Na_2_HPO_4_(0.6 mM), MgSO_4_-7H_2_O (1.2 mM), NaHCO_3_ (12 mM), KHCO_3_ (10 mM), HEPES Na Salt (0.922 mM), Taurine (30 mM), BDM (10 mM), and Glucose (5.5 mM). After 5 min of continuous perfusion at 37°C, hearts were lysed using liberase (5 mg/mL, Roche) for 3 min. Subsequently, hearts were sequentially incubated with isolation buffer mixed with 10% FBS and 200, 400 or 900 µM CaCl_2_ for 10 min each. The final pellet was re-suspended in M199 solution supplemented with Penicillin/Streptomycin (100 IU/mL), L-carnitine (2 mM), creatine (5 mM), taurine (5 mM), and blebbistatin (25 µM).^30^

Adult murine ventricular cardiomyocytes were subjected to a SIRI protocol of 30 min simulated ischaemia [comprising ‘ischaemic’ buffer containing KH_2_PO_4_ (0.5 mM), NaHCO_3_ (5 mM), MgCl_2_._6_H_2_O (0.6 mM), Na Hepes (12.5 mM), NaCl (74 mM), KCl (16 mM), Na-Lactate (20 mM), and CaCl_2_ (1.26 mM) at pH 6.2 in a sealed hypoxic chamber] and 15 min simulated reperfusion [by removing the cells from the hypoxic chamber and placing them in ‘normoxic’ buffer containing KH_2_PO_4_ (0.5 mM), NaHCO_3_ (5 mM), MgCl_2_.6H_2_O (0.6 mM), Na Hepes (12.5 mM), NaCl (97.60 mM), KCl (2.9 mM), D-Glucose (10 mM), Na-Pyruvate (2 mM), and CaCl_2_ (1.26 mM) at pH 7.4].

Cells were randomised to receive the following treatment protocols: (i) normoxic time control: cells were incubated in ‘normoxic’ buffer for 60 min; (ii) vehicle control: cells were placed in normoxic buffer containing the distilled water vehicle control for 15 min, prior to the SIRI protocol; and (iii) hydralazine pre-treatment: cells were placed in normoxic buffer containing hydralazine (1 µM) for 15 min, which was then replaced with normoxic buffer alone for a further 15 min, prior to the SIRI protocol.

Following the SIRI protocol, cell death was assessed using 1 µg/mL of propidium iodide solution. Five randomly selected images comprising ∼90 cells per image were acquired using a 10× dry objective of a Leica DMi8 microscope (*N*=5 mice per treatment group). Using a Nikon A1 confocal equipped with a live cell humidity-controlled imaging chamber at 37°C (5% CO_2_), mitochondrial morphology following SIRI was assessed by analysing the mitochondrial Dendra2 green fluorescent signal in randomly imaged cells (24 cells per each *N*). Cells were assigned as showing mainly mitochondrial elongation (>50%) or fragmentation (>50%) by an experienced observer (S.B.K.) blinded to the treatment allocation.

Mitochondrial fusion events at baseline and following SIRI were assessed using a Nikon A1 confocal equipped with a live cell humidity-controlled imaging chamber at 37°C (5% CO_2_) (*N*=5 mice per treatment group). For each cell, three regions of interest (1.24 µm^2^) containing a single mitochondrion from each of the three mitochondrial subpopulations [intermyofibrillar (IMF) mitochondria, subsarcolemmal mitochondria (SSM) or perinuclear mitochondria (PNM)] were randomly selected and photo-activated using a 405 nm laser to photo-switch mitochondrial Dendra2 protein from green to red fluorescent signal. A mitochondrial fusion event was defined as the propagation of red fluorescent signal from the photo-activated mitochondrion to an adjacent mitochondrion. For each treatment group, 20 cells were imaged at 4 min intervals for a total of 16 min, and the total number of mitochondrial fusion events was counted. Images were aligned using the TurboReg plugin of ImageJ.

### 2.6 Isolated perfused *ex vivo* murine heart IRI model

The effect of hydralazine on MI size was investigated using an isolated perfused *ex vivo* murine heart IRI model.^31^^,^^32^ Mice were anaesthetised via intraperitoneal injection of pentobarbitone sodium solution at a final dose of 0.2–0.4 g/kg body weight. Anticoagulant heparin sodium (Rockhardt UK Ltd, Wrexham, UK) at a dose of up to 5000 units/kg body weight was co-administered with the anaesthetic. Upon the onset of deep anaesthesia, identified as the loss of the pedal pain withdrawal reflex, slowing of heart rate and breathing, mice were euthanized by excising the heart, and immediately securing the heart onto the cannula of a Langendorff-perfusion apparatus (in constant flow-mode) and perfused with Krebs–Henseleit buffer containing NaCl (118.5 mM), NaHCO_3_ (25 mM), d-glucose (11 mM), KCl (4.7 mM), MgSO_4_ (1.2 mM), KH_2_PO_4_ (1.2 mM), and CaCl_2_ (1.8 mM), bubbled with 5% CO_2_ and 95% O_2_. The temperature of the hearts was maintained between 36 and 38°C and monitored using a fine thermocouple [TM Electronics (UK) LTD, Goring by Sea, UK] retrogradely passed into the right ventricular outflow tract via the pulmonary artery. Hearts were initially stabilized for 15 min, and were then randomly perfused with either normal saline vehicle control or hydralazine (1 µM) for 15 min prior to 35 min of global ischaemia followed by 60 min of reperfusion (with vehicle control or hydralazine present during the reperfusion period). Hearts were then frozen, and infarct size determined by tetrazolium staining (see Section 2.7 for method).

### 2.7 Acute myocardial *in vivo* murine IRI model

The effect of hydralazine administered at the onset of reperfusion on MI size was investigated using a murine acute myocardial *in vivo* IRI model.^33^^,^^34^ Mice were intubated under general anaesthesia (65 mg/kg ketamine, 13 mg/kg xylazine, i.p.) and analgesia (0.1 mg/kg Buprenorphine). The animals received mechanical ventilation (VentElite, Harvard apparatus) at a frequency of 140 breaths/min and tidal volume of 140–160 µL. If necessary, 0.5% isoflurane was used during the procedure to assure proper anaesthetisation. After shaving and proper disinfection, thoracotomy was performed between the 3rd and 4th left intercostal space as previously described. After removing the pericardium, the left anterior descending coronary artery (LAD) was visualised and ligatured over a silicon tube with a 7–0 silk suture. Myocardial ischaemia was confirmed by visual cyanosis. The mice received either hydralazine (10 mg/kg) or normal saline vehicle control, as an intravenous bolus, 3 min before reperfusion through the tail vein. After 60 min, the ligation was released, and the heart was reperfused for 120 minutes. Reperfusion was confirmed by the colour change in the ventricular surface.

Infarct size was measured as reported previously.^33^^,^^34^ Briefly, after 120 min of reperfusion, the LAD was re-ligated at the original site, and under deep anaesthesia (identified as the loss of the pedal pain withdrawal reflex, slowing of heart rate and breathing) the mice were euthanised by excising the heart, and the area-at-risk (AAR) was delineated by perfusion with 5% Evans blue dye. The hearts were then quickly frozen and cut into ∼1 mm thick transverse slices from base to apex. The slices were incubated in 1% triphenyltetrazolium chloride in sodium phosphate buffer (pH 7.4) at 37°C for 20 min. They were then fixed in 10% formalin and placed between two glass slides, and the infarct size and AAR were quantified using ImageJ. Infarct size was expressed as a % of the AAR (IS/AAR%).

### 2.8 Statistics

Graph Pad Prism and Microsoft Excel were used to plot the graphs and perform the statistical analysis. One-way ANOVA and *t*-test were used to assess the differences between treatment groups. *P*-value ≤0.05 was considered significant.

## 3. Results

### 3.1 Hydralazine binds to human Drp1 and inhibits its GTPase activity

**Figure 1:**
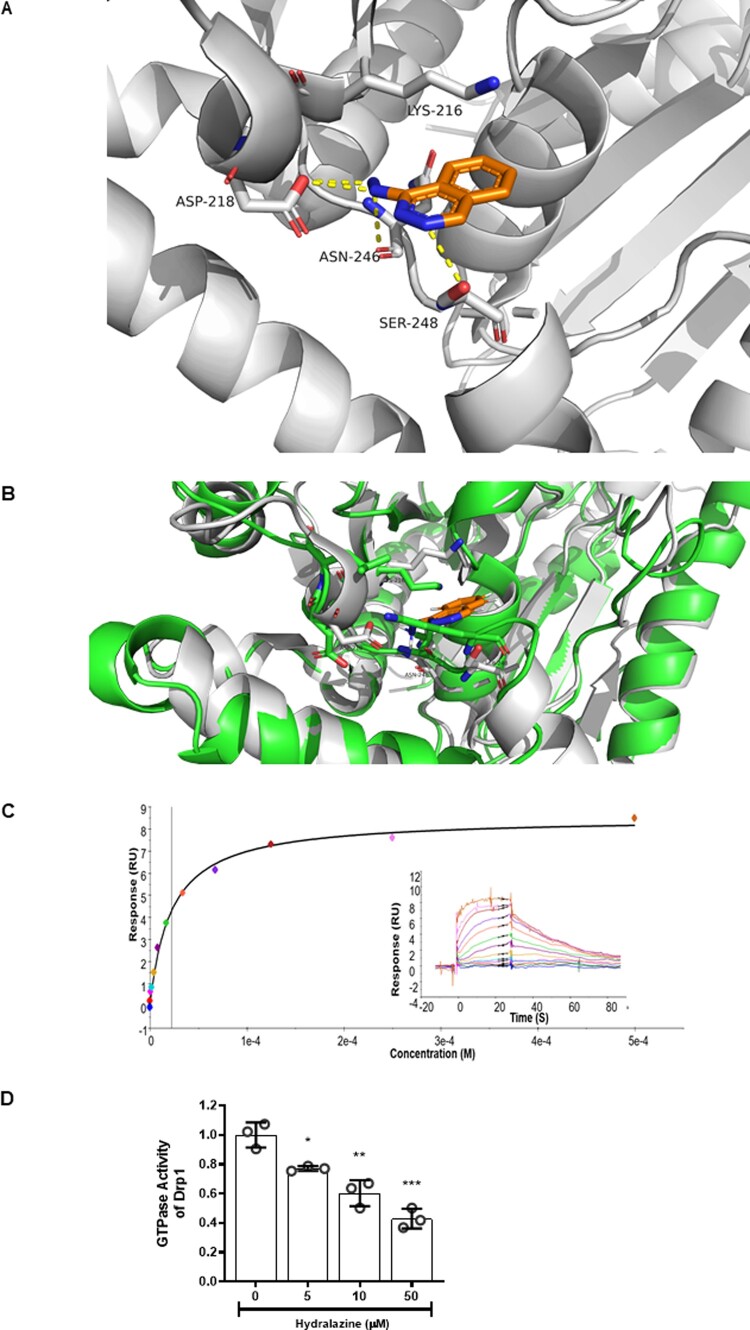
Hydralazine putatively binds to Drp1 and inhibits its GTPase activity. (*A*) Molecular docking studies demonstrated that hydralazine (orange structure) putatively binds to the GTPase domain of Drp1 (white structure) via hydrogen bonds (yellow dashed lines) to Asp218, Asn246, and Ser248. Lys216 is able to charge-pi stack to the phthalazine ring of hydralazine, thereby stabilizing its binding to Drp1. (*B*) Overlay of Drp1 (white) with OPA1 (green) showing that the residues, which we propose are important for the interaction between hydralazine to Drp1 (Lys216, Asn218, Asn246, and Ser248, equivalent to Lys469, Asp470, Thr503, and Lys505 in OPA1) are not present in OPA1. Specifically, the Drp1 Ser248 to OPA1 Lys505 change blocks this binding pocket, thereby preventing hydralazine (orange) from binding to the GTPase domain of OPA1. (*C*) A representative SPR (Biacore T200) experiment demonstrating a direct dose–response binding interaction between the bound recombinant Drp1 protein exposed to increasing concentrations of hydralazine. Each experiment was run in duplicate. Overall this interaction had a calculated KD of 8.6±1.0 µM. *N*=3 independent experiments. (*D*) Hydralazine inhibited GTPase activity of Drp1 in a dose-dependent manner. *N*=3 independent experiments. Statistical analysis was performed using one-way ANOVA with Tukey’s multiple comparison post-test. Data are expressed as mean±SEM. *P<0.05, **P<0.01, ***P<0.001, vs. control (Drp1 alone).

The molecular docking studies suggested that hydralazine could bind to the GTPase domain of Drp1 via putative hydrogen bonds to the Asp218, Asn246, and Ser248 residues of Drp1 (*Figure 1A*). In addition, the Lys216 residue of Drp1 had a putative charge-π stack to the phthalazine ring of hydralazine, thereby stabilizing binding to the GTPase domain of Drp1. We found that hydralazine was unable to dock with OPA1 in the same manner as it did to Drp1. Specifically, the residues, which we propose are important for the interaction between hydralazine and Drp1 (Lys216, Asn218, Asn246, and Ser248, equivalent to Lys469, Asp470, Thr503, and Lys505 in OPA1) are not fully conserved in OPA1. Specifically, the Drp1 Ser248 to OPA1 Lys505 change blocks this binding pocket, thereby preventing hydralazine from binding to the GTPase domain of OPA1 (*Figure 1B*).

The ability of hydralazine to directly bind to Drp1 was confirmed by SPR. Hydralazine directly bound to recombinant Drp1 protein with a binding constant (KD) of 8.6±1.0 µM (*Figure 1C*). Hydralazine was also shown to significantly inhibit the GTPase activity of recombinant Drp1 protein in a dose-dependent manner (*Figure 1D*). Overall, these data suggest that hydralazine can directly bind to Drp1, and inhibit its GTPase activity.

### 3.2 Hydralazine prevented H_2_O_2_-induced mitochondrial fragmentation and depolarization

**Figure 2:**
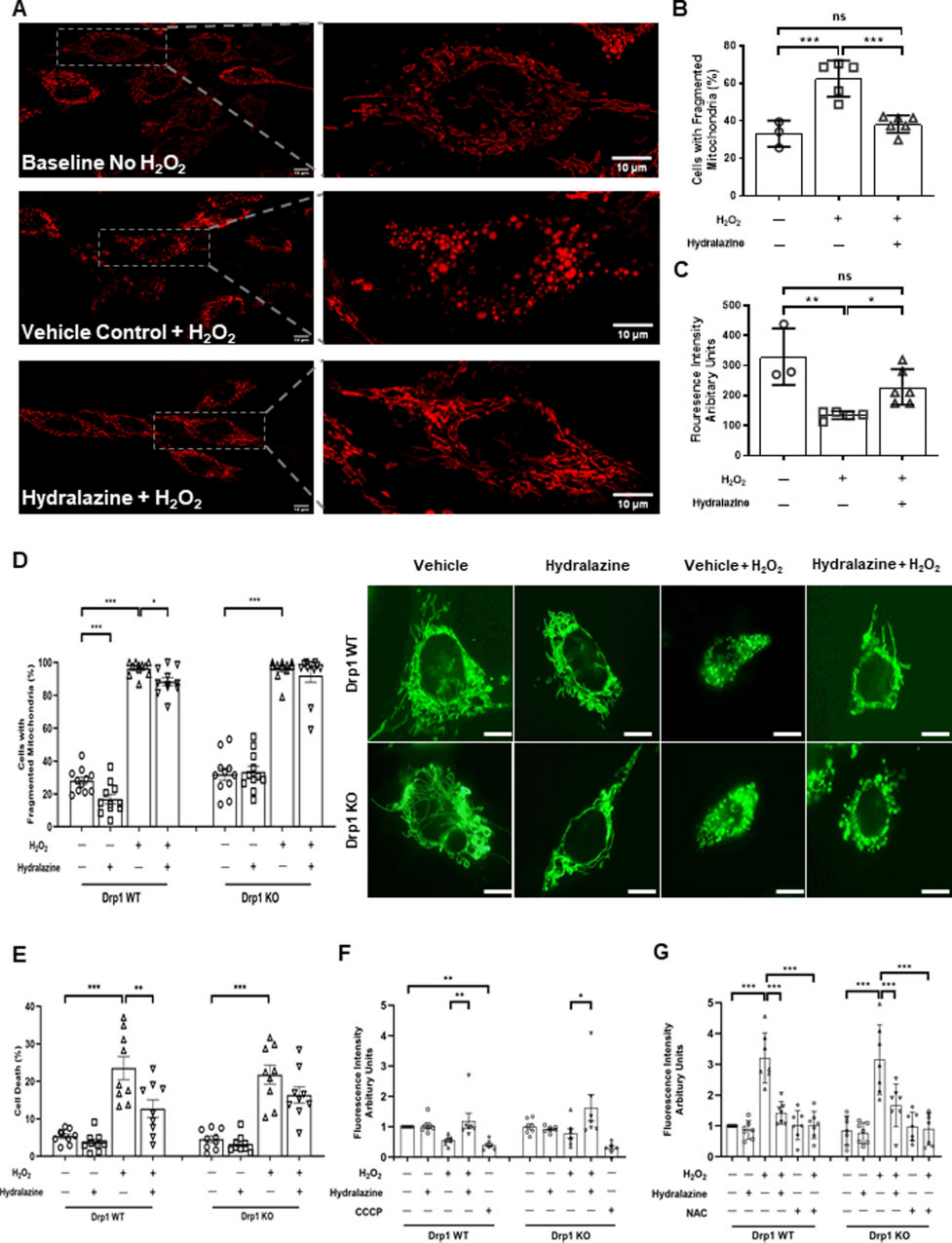
Hydralazine inhibited mitochondrial fragmentation and depolarization induced by oxidative stress. (*A*) Representative confocal images of HeLa cells showing mitochondrial fragmentation in induced by H_2_O_2_, an effect which was attenuated in cells pre-treated with hydralazine. Scale bars are 10 µm. (*B*) HeLa cells subjected to H_2_O_2_ displayed more mitochondrial fragmentation when compared to baseline cells (no H_2_O_2_), and this effect was attenuated in cells pre-treated with hydralazine. Statistical analysis was performed using one-way ANOVA with Tukey’s multiple comparison post-test. Data are expressed as mean±EM. ***P=0.0002. Baseline (no H_2_O_2_), *N*=3 independent experiments. Vehicle control+H_2_O_2_, *N*=5 independent experiments. Hydralazine+H_2_O_2_, *N*=6 independent experiments. (*C*) H_2_O_2_ induced mitochondrial membrane depolarization in HeLa cells, when compared to baseline cells (no H_2_O_2_), and this effect was attenuated in cells pre-treated with hydralazine. Statistical analysis was performed using one-way ANOVA with Tukey’s multiple comparison post-test. Data are expressed as mean±SEM. *P<0.05. **P<0.01. Baseline (no H_2_O_2_), *N*=3 independent experiments. Vehicle control+H_2_O_2_, *N*=5 independent experiments. Hydralazine+H_2_O_2_, *N*=6 independent experiments. (*D*) Representative confocal images of WT and Drp1 KO MEFs. Under baseline conditions (no H_2_O_2_), pre-treatment with hydralazine reduced mitochondrial fragmentation in WT MEFs but not in Drp1 KO MEFs. In response to H_2_O_2_, mitochondrial fragmentation was increased in both WT and Drp1 KO MEFs, and hydralazine attenuated mitochondrial fragmentation in WT MEFS, but not in Drp1 KO MEFs. Statistical analysis was performed using one-way ANOVA with Sidak’s multiple comparison post-test. Data are expressed as mean±SEM. *N*=11 independent experiments. For Drp1 WT: *P=0.0445. **P=0.0031 for vehicle control no H_2_O_2_ vs. hydralazine no H_2_O_2_ and ***P<0.0001 for vehicle control no H_2_O_2_ vs. vehicle control+H_2_O_2_. For Drp1 KO: ***P<0.0001. Scale bars are 10 µm. (*E*) H_2_O_2_ induced cell death in both WT and Drp1 KO MEFs, and hydralazine significantly attenuated cell death in WT MEFs but not in Drp1 KO MEFs. Statistical analysis was performed using one-way ANOVA with Sidak’s multiple comparison post-test. *N* =9 independent experiments. Data are expressed as mean±SEM. For Drp1 WT: ***P<0.0001. **P<0.0018. For Drp1 KO. ***P<0.0001. (*F*) H_2_O_2_ induced mitochondrial membrane depolarization in both WT and Drp1 KO MEFs, and this effect was attenuated in cells pre-treated with hydralazine. CCCP was used as a positive control to induce mitochondrial membrane depolarization. Statistical analysis was performed using one-way ANOVA with Sidak’s multiple comparison post-test. *N*=7 independent experiments. Data are expressed as mean±SEM. For Drp1 WT: **P=0.0047 vehicle control+H_2_O_2_ vs. hydralazine+H_2_O_2_ and **P=0.0081 for vehicle control no H_2_O_2_ vs. CCCP. For Drp1 KO: *P=0.0337. (*G*) H_2_O_2_ induced the formation of mitochondrial ROS in both WT and Drp1 KO MEFs, and this effect was attenuated in cells pre-treated with hydralazine. N-acetyl cysteine (NAC) was used as a positive control for scavenging ROS. Statistical analysis was performed using one-way ANOVA with Sidak’s multiple comparison post-test. *N*=7 independent experiments. Data are expressed as mean±SEM. For Drp1 WT: ***P<0.0001 vehicle control no H_2_O_2_ vs. vehicle control+H_2_O_2_ and ***P<0.0001 for vehicle control+H_2_O_2_ vs. hydralazine+H_2_O_2_ and ***P<0.0001 vehicle control+H_2_O_2_ vs. NAC+H_2_O_2_. For Drp1 KO: ***P<0.0001 vehicle control no H_2_O_2_ vs. vehicle control+H_2_O_2_ and ***P=0.0008 for vehicle control+H_2_O_2_ vs. hydralazine+H_2_O_2_ and ***P<0.0001 vehicle control+H_2_O_2_ vs. NAC+H_2_O_2_.

Given that hydralazine was able to bind to Drp1 activity and inhibit its GTPase activity, we next investigated whether hydralazine could inhibit mitochondrial fission and mitochondrial membrane depolarization induced by oxidative stress in HeLa cells. Compared to baseline cells (not treated with H_2_O_2_), treatment with H_2_O_2_ increased the proportion of cells displaying mitochondrial fragmentation. This effect was attenuated with hydralazine pre-treatment compared to vehicle control (baseline no H_2_O_2_ 33.1±4.0% vs. vehicle control+H_2_O_2_ 62.5±4.3% vs. hydralazine+H_2_O_2_ 38.2±1.9%, *P=*0.0002) (*Figure 2A and B*). Compared to baseline cells (not treated with H_2_O_2_), treatment with H_2_O_2_ decreased the mitochondrial membrane potential. This effect was attenuated with hydralazine pre-treatment compared to vehicle control [baseline no H_2_O_2_ 329.0±54.5 arbitrary units (au) vs. vehicle control+H_2_O_2_ 134.6±6.0 au vs. hydralazine 228.3±24.1 au, *P=*0.0023] (*Figure 2C*).

To assess whether the effects of hydralazine on mitochondrial function were Drp1-dependant, we evaluated the effects of hydralazine in Drp1 WT and Drp1 KO MEFs. Hydralazine pre-treatment reduced mitochondrial fragmentation under both baseline conditions (vehicle control 28.1 ±2.2% vs. hydralazine 16.9±2.8%, *P*=0.0031) (*Figure 2D*), and in response to H_2_O_2_ treatment (vehicle control 96.4±1.3% vs. hydralazine 88.4±2.4%, *P*=0.0445) (*Figure 2D*), and both these effects of hydralazine were attenuated in Drp1 KO MEFs (*Figure 2D*). Pre-treatment with hydralazine reduced cell death in response to H_2_O_2_ treatment in Drp1 WT MEFs (vehicle control 23.6±3.1% vs. hydralazine+H_2_O_2_ 12.7±2.3%, *P*=0.0018 (*Figure 2E*), but this protective effect of hydralazine was attenuated in Drp1 KO MEFs (*Figure 2E*). These results suggest that the effects of hydralazine on inhibiting H_2_O_2_-induced mitochondrial fission and cell death are dependent on Drp1. Pre-treatment of hydralazine had no effect on either mitochondrial membrane potential or mitochondrial formation of ROS under baseline conditions in either the WT or Drp1 KO MEFS (*Figure 2F and G*). Hydralazine did preserve H_2_O_2_-induced mitochondrial membrane depolarization (vehicle control 0.8±1.5 au vs. hydralazine+H_2_O_2_ 1.6±0.4 au, *P*=0.0337 (*Figure 2F*) and attenuated mitochondrial production of ROS (vehicle control 3.2±0.3 au vs. hydralazine 1.4±0.1 au, *P*=0.0001) (*Figure 2G*). Interestingly, both these effects of hydralazine appeared to be independent of Drp1, as they were still present in the Drp1 KO MEFs (*Figure 2F and G*).

### 3.3 Hydralazine inhibited mitochondrial fission and prevented cell death following SIRI

**Figure 3:**
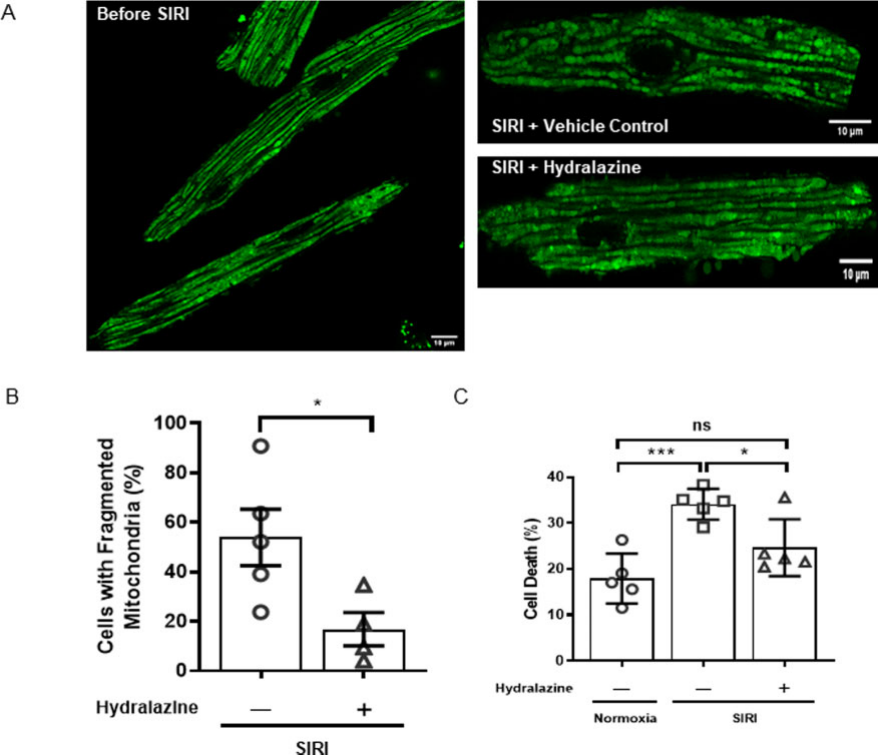
Hydralazine inhibited mitochondrial fragmentation and reduced cell death following SIRI. (*A*) Representative confocal images of adult murine ventricular cardiomyocytes subjected to SIRI showed that treatment with hydralazine inhibits IRI-induced mitochondrial fragmentation when compared to vehicle control. Scale bars are 10 µm. (*B*) Treatment of adult murine ventricular cardiomyocytes subjected to SIRI with hydralazine reduced the mean percentage of cells with fragmented mitochondria, when compared to vehicle control. Statistical analysis was performed using the Student’s *t*-test. Data are expressed as mean±SEM. *P=0.034. *N*=4–5 independent experiments. (*C*) Treatment of adult murine ventricular cardiomyocytes subjected to SIRI with hydralazine reduced cell death (as evidenced by propidium iodide positive cells), when compared to vehicle control. Statistical analysis was performed using one-way ANOVA with Tukey’s multiple comparison post-test. Data are expressed as mean±SEM. ^*^P<0.05. ***P<0.001. *N*=5 independent experiments.

Having demonstrated that hydralazine inhibited mitochondrial fission in HeLa cells and MEFs subjected to oxidative stress, we next investigated whether hydralazine could inhibit mitochondrial fission induced by SIRI in adult murine ventricular cardiomyocytes. Under basal conditions, there was no mitochondrial fragmentation in adult mouse cardiomyocytes (*Figure 3A*). However, in response to SIRI, adult cardiac mitochondria underwent fragmentation (*Figure 3A*), such that the proportion of cells showing mitochondrial fragmentation increased after SIRI, and this effect was attenuated by pre-treatment with hydralazine (vehicle control 53.9±11.4% vs. hydralazine 16.9±6.7%, *P*=0.034) (*Figure 3A and B*). Pre-treatment of cells with hydralazine also reduced cell death following SIRI when compared to vehicle control (normoxic time control 18.0±2.4% vs. vehicle control 34.1±1.5% vs. hydralazine 24.7±2.8%, *P*=0.0012) (*Figure 3C*). These findings suggest that pre-treatment with hydralazine can inhibit mitochondrial fission and prevent cell death in adult cardiomyocytes following SIRI.

### 3.4 Hydralazine-preserved mitochondrial fusion events following SIRI

**Figure 4:**
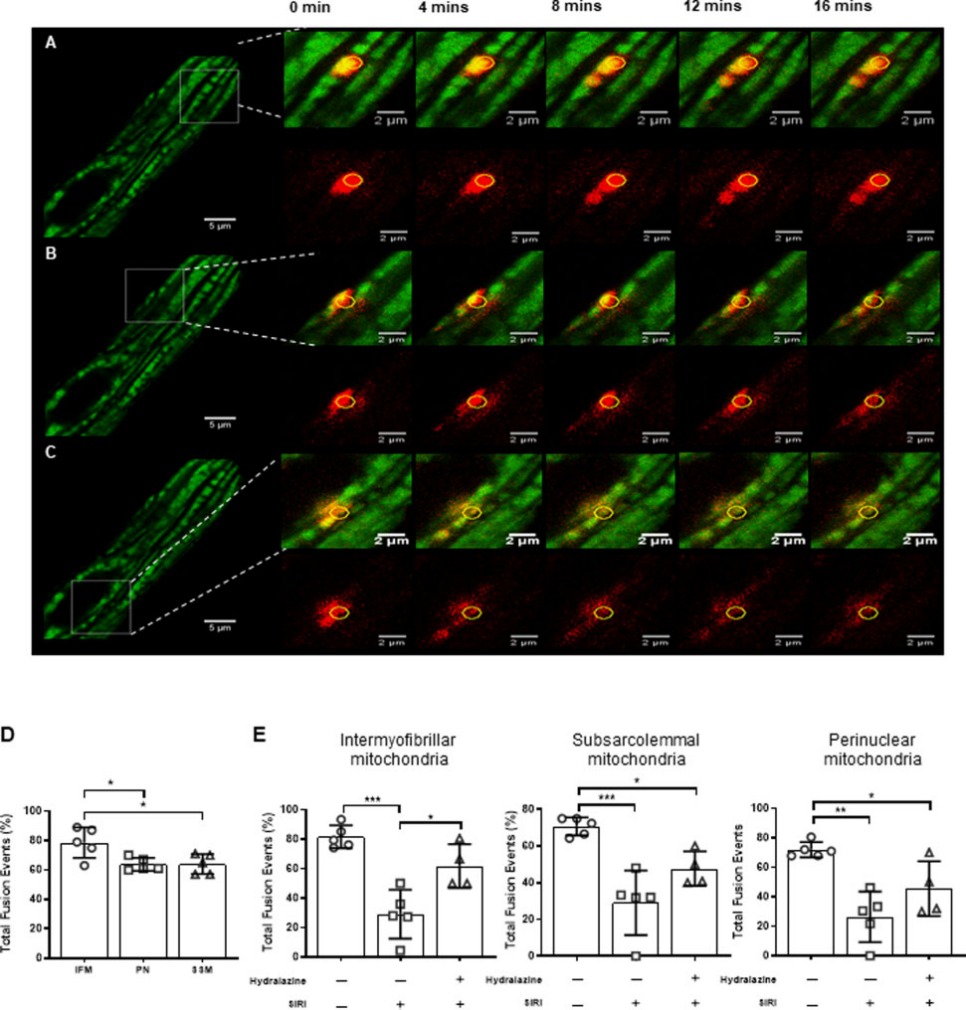
Hydralazine-preserved mitochondrial fusion in adult murine ventricular cardiomyocyte mitochondria subjected to SIRI. Representative images of a single adult murine ventricular cardiomyocyte showing propagation of photo-switchable green to red fluorescent mitochondrial Dendra2 protein to neighbouring mitochondria from the region of interest (yellow circle) following SIRI in (*A*) IMF mitochondria, (*B*) SSM, and (*C*) PNM. Scale bars are 5 µm left panels, and inset images scales bars are 2 µm. (*D*) Quantification of mitochondrial fusion events at baseline in the three different mitochondrial subpopulations showed an increased number of mitochondrial fusion events in IMF mitochondria compared to SSM and PNM. Statistical analysis was performed using one-way ANOVA with Tukey’s multiple comparison post-test. *P<0.05. *N* =5 independent experiments. (*E*) Quantification of mitochondrial fusion events following SIRI in vehicle control in the three different mitochondrial subpopulations. SIRI is shown to reduce mitochondrial fusion events and this effect was attenuated in the presence of hydralazine. Statistical analysis was performed using one-way ANOVA with Tukey’s multiple comparison post-test. Data are expressed as mean±SEM. *P<0.05, **P<0.01 and ***P<0.001. *N*=4–5 independent experiments.

In the Section 3.3, we found that hydralazine inhibited mitochondrial fission following SIRI in adult mouse cardiomyocytes. We next investigated whether hydralazine can preserve mitochondrial fusion events following SIRI. Mitochondrial fusion events were defined as the propagation of photo-switched mitochondrial Dendra2 green to RFP from the original ROI of photoactivation, to adjacent mitochondria (*Figure 4 A–C*). Under baseline conditions, the number of mitochondrial fusion events was higher in IMF mitochondria when compared to SSM and PNM (IMF mitochondria 78.6±4.7% vs. SSM 64.0±3.0% vs. PNM 64.0±2.0%, *P*=0.014) (*Figure 4D*). The mitochondrial fusion events were observed to occur in one direction, in a longitudinal orientation, and in line with the myofibrils. In response to SIRI, the number of mitochondrial of fusion events was significantly decreased in all three mitochondrial subpopulations (*Figure 4E*), and this effect was significantly attenuated in IMF mitochondria of cells pre-treated with hydralazine (normoxic time control: 78.6±4.7% vs. vehicle control 29.1±7.4% vs. hydralazine 61.8±7.4%, *P*=0.0002) (*Figure 4E*). In SSM and PNM, the effect of hydralazine on preserving mitochondrial fusion events was not significant. These findings suggest that pre-treatment with hydralazine can preserve mitochondrial fusion events in adult cardiomyocytes following SIRI.

### 3.5 Hydralazine reduces MI size in *ex vivo* and *in vivo* murine hearts subjected to IRI

**Figure 5:**
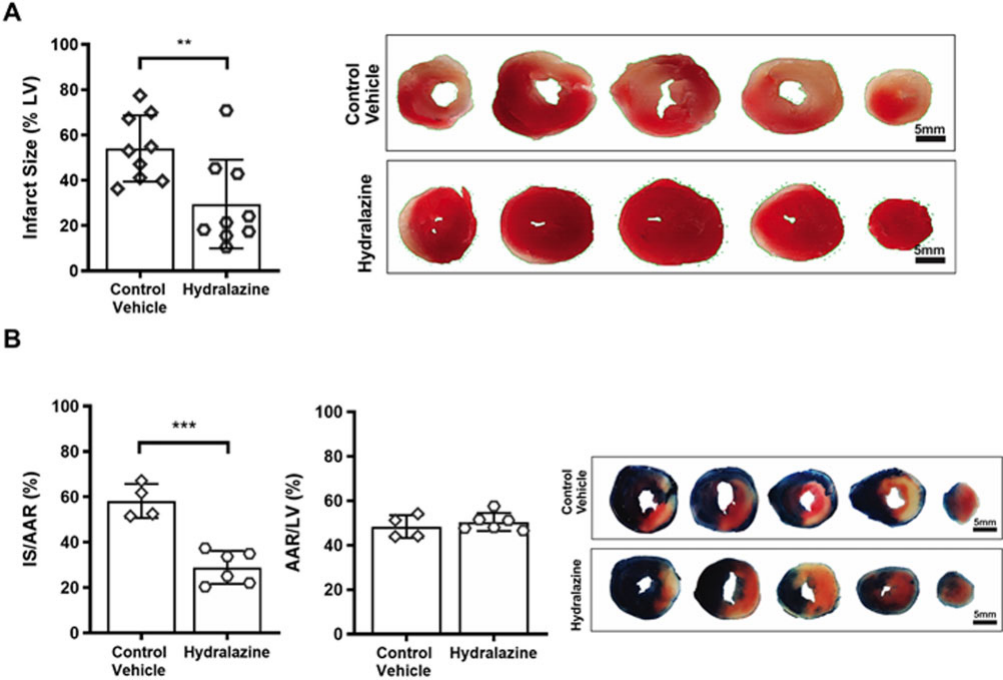
Hydralazine reduced *ex vivo* and *in vivo* MI size. (*A*) When compared to vehicle control (*N*=9), pre-treatment with hydralazine (*N*=9) reduced MI size expressed as % of left ventricular volume in the isolated perfused murine hearts subjected to global acute myocardial IRI. The top panel depicts representative images of a murine heart slices treated with either control vehicle or hydralazine. Scale bars are 5 mm. (*B*) When compared to control vehicle (*N*=4), treatment with hydralazine at the onset of reperfusion (*N*=6) reduced MI size expressed as a % of the AAR in the murine hearts subjected to *in vivo* regional acute myocardial IRI. The centre panel shows that the AAR was not different between treatment groups. The right panel depicts representative images of a murine heart slices treated with either control vehicle or hydralazine. Statistical analysis was performed using the Student’s *t*-test. Data are expressed as mean±SEM. **P=0.0083. ***P=0.0003. Scale bars are 5 mm.

Having found that hydralazine inhibited mitochondrial fission, preserved mitochondrial fusion, and reduced cell death in adult murine cardiomyocytes following SIRI, we next investigated whether treatment with hydralazine could reduce MI size. Treatment with hydralazine prior to ischaemia and during reperfusion reduced MI size in the isolated *ex vivo* perfused murine hearts subjected to acute IRI, when compared to vehicle control (hydralazine 29.6±6.5% vs. vehicle control 54.1±4.9%, *P*=0.0083) (*Figure 5A*). Similarly, hydralazine administered immediately prior to reperfusion reduced MI size (as a % of the AAR) in the *in vivo* murine hearts subjected to acute IRI, when compared to vehicle control (hydralazine 28.9±2.9% vs. vehicle control 58.2±3.7%, *P*=0.0003) (*Figure 5B*). There was no difference in the size of the AAR between the treatment groups (*Figure 5B*). These data suggest that treatment with hydralazine either prior or at the onset of reperfusion can reduce MI size in the *ex vivo* and *in vivo* murine hearts subjected to acute IRI.

## 4. Discussion

In this study, we report for the first time that the acute administration of hydralazine protected the heart against IRI by inhibiting Drp1-mediated mitochondrial fission, highlighting a novel mechanism underlying hydralazine-mediated acute cardioprotection. We report for the first time that hydralazine binds to the mitochondrial fission protein, Drp1, and inhibits its GTPase activity, the latter of which is required for Drp1 to mediate mitochondrial fission.^9^ Through this action, hydralazine inhibited mitochondrial fission and cell death induced by oxidative stress, suggesting a link between the hydralazine-induced preservation of mitochondrial dynamics and cytoprotection. Next, we demonstrated that hydralazine inhibited mitochondrial fission, preserved mitochondrial fusion events, and reduced cell death in isolated adult murine ventricular cardiomyocytes subjected to SIRI, suggesting a direct cytoprotective effect of hydralazine. Following this, we found that treatment with hydralazine both prior to ischaemia and at reperfusion reduced MI size in the isolated perfused *ex vivo* murine hearts subjected to acute IRI. Finally, we demonstrated that the acute administration of hydralazine, at the onset of reperfusion, reduced MI size in the murine model of *in vivo* acute IRI, confirming the clinical applicability of this cardioprotective approach to AMI patients.

In our study, we used adult mice expressing the photo-switchable mitochondrial protein, Dendra2, to demonstrate in isolated ventricular cardiomyocytes that mitochondria undergo fission in response to SIRI, and that hydralazine prevented IRI-induced Drp1-mediated mitochondrial fission. Using the same Dendra2 mice, we also showed that mitochondrial fusion events, documented by the propagation of photo-switched red fluorescent Dendra protein between adjacent mitochondria, take place under basal conditions, and these occurred more frequently in IMF mitochondria (when compared to SSM and PNM). The reason for this is not clear, but it could relate to its specific role of IMF mitochondria in generating ATP to maintain normal cardiac contractile function. Importantly, we demonstrated that IRI-reduced mitochondrial fusion events in all three subpopulations of mitochondria (a finding, which correlated with the observed increase in mitochondrial fragmentation with IRI). This effect was attenuated by treatment with hydralazine, confirming the protective effect of the latter on mitochondrial morphology. Although other studies have used mitochondria-targeted photoactivatable GFP to track mitochondrial fusion events in adult rodent cardiomyocytes,^35^^,^^36^ our study is the first to use mitochondrial Dendra2 mice for this purpose, and the first to study this phenomenon in the context of acute myocardial IRI and cardioprotection.

Hydralazine is a Food and Drug Administration (FDA)-approved therapy for treating essential hypertension, severe hypertension in pregnancy,^16^ and when used in combination with isosorbide-dinitrate, it provides a treatment option for symptomatic patients with chronic HF due to reduced ejection fraction, who cannot tolerate ACE-I/ARB therapy (reviewed in the Reference^17^). The known actions of hydralazine related to these conditions are to relax vascular smooth muscle cells (VSMCs) and induce arteriolar dilatation, resulting in a lowering of total peripheral resistance, and a reduction in myocardial workload. However, the mechanisms through which hydralazine confers this vasorelaxation effect, and confers a benefit in HF remain unclear, and have been attributed to the opening of high conductance Ca^2+^-activated K^+^ channels in VSMCs,^37^ inhibiting the inositol 1,4,5 triphosphate-induced release of Ca^2+^ from the sarcoplasmic reticulum (SR) in VSMCs^38^; reducing SR leak, improving SR Ca^2+^ reuptake, and restoring SR Ca^2+^ content^39^; and production of cGMP in VSMCs.^40^ Interestingly, hydralazine has also been reported to have several non-vasorelaxation effects including: decreasing vascular ROS production thereby preventing nitrate intolerance^41^; stabilising hypoxia-inducible factor-1α in endothelial cells and stimulating angiogenesis via VEGF^42^; protection against acute renal IRI^43^^,^^44^; and prevention of renal fibrosis in a kidney injury model.^45^

A limited number of experimental studies have suggested a cardioprotective effect with hydralazine in rat models of acute myocardial IRI, although the mechanisms underlying this effect have not been clearly elucidated. In these studies, they have mainly been attributed to non-specific anti-apoptotic, anti-inflammatory and antioxidant effects of hydralazine,^18^^,^^19^ with one recently published study reporting that hydralazine administered either prior to ischaemia or reperfusion activated the known cardioprotective PI3K-Akt pathway.^19^ In our study, we have shown that in addition to these cardioprotective effects, hydralazine confers mitochondrial protection by inhibiting mitochondrial fission. Mitochondria have been demonstrated to undergo Drp1-mediated fission in response to acute IRI, resulting in mitochondrial dysfunction and cell death.^6^^,^^7^^,^^10^^,^^11^^,^^13^^,^^27^^,^^46^ The importance of mitochondrial fission as a therapeutic target for cardioprotection has been established by the findings that genetic or pharmacological inhibition of IRI-induced fission confers cardioprotection in cardiomyocyte models of SIRI, and in rodent models of acute myocardial IRI.^6^^,^^7^^,^^10^^,^^11^^,^^13^^,^^27^^,^^46^

However, the majority of these studies have used the putative Drp1 inhibitor, Mdivi-1,^12^ to suppress IRI-induced mitochondrial fission and confer cardioprotection.^7^^,^^11^^,^^27^^,^^46^ Recent studies suggest that mdivi-1 has off-target effects that are independent of its inhibitory effects on Drp1 GTPase activity, including it being a weak and reversible inhibitor of complex I, modulating mitochondrial production of ROS in neurons,^14^ inhibiting complex II, and modulating transient opening of the mitochondrial permeability transition pore in isolated adult cardiomyocytes.^15^ Furthermore, in a previously published small pilot study,^47^ we failed to demonstrate cardioprotection with mdivi-1 administered at reperfusion in a pig model of acute myocardial IRI, although this could have been due to the small sample size and insufficient dosing. As such, there is a need for novel more specific inhibitors of Drp1 to provide more effective cardioprotection, and facilitate the clinical translation of acute mitochondrial fission inhibition as a cardioprotective strategy.^48^^,^^49^ In this regard, our discovery that the FDA-approved drug, hydralazine (which is already used in clinical practice for cardiovascular disease), can also confer cardioprotection by inhibiting Drp-1 mediating mitochondrial fission, raises the possibility of repurposing an existing FDA-approved drug for potentially improving cardiovascular outcomes in AMI patients. However, before this approach can be tested in the clinical setting, dosing studies with hydralazine will have to be performed in a large animal acute myocardial IRI model to establish the optimal dose, which confers cardioprotection without causing any significant haemodynamic effects, such as lowering blood pressure and inducing reflex tachycardia.

A potential limitation of our study includes the use of female mice in the *ex vivo* IRI studies, and male mice in the *in vivo* IRI experiments, although on the other hand, this could be seen as an advantage as it demonstrates that hydralazine confers cardioprotection in both female and male mice, thereby highlighting the translational potential of hydralazine. Also, given that hydralazine has been shown to confer cardioprotection through different mechanisms including non-specific anti-apoptotic, anti-inflammatory and antioxidant effects,^18^^,^^19^ we cannot exclude that these actions of hydralazine did not contribute to the cardioprotective effects observed in our study. This is evident in our study by the fact that the effects of hydralazine in attenuating H_2_O_2_-induced mitochondrial ROS formation and mitochondrial membrane depolarization were present in Drp1 KO MEFs, suggesting that these effects of hydralazine were independent of Drp1. However, the effects of hydralazine in reducing H_2_O_2_-induced mitochondrial fragmentation and cell death were absent in Drp1 KO MEFs, suggesting that these effects of hydralazine were dependent on Drp1.

In summary, we provide evidence that in addition to its known anti-apoptotic, anti-inflammatory and antioxidant effects, hydralazine confers acute cardioprotection by inhibiting IRI-induced mitochondrial fission. These findings open up the possibility of repurposing hydralazine as a novel cardioprotective agent for patients with AMI.

## Authors’ contributions

All authors made substantial contributions to the conception or design of the work; or the acquisition, analysis, or interpretation of data for the work; and drafted the work or revised it critically for important intellectual content; and gave final approval of the version to be published.

**Conflict of interest**: none declared.

## Funding

This work was funded in part by Stafford Fox Medical Research Foundation (to S.Y.L.) and infrastructure funding from the Victorian Government (Australia) Operational Infrastructure Support Scheme to St Vincent’s Institute of Medical Research. J.K.H. is a 5 Point Foundation Christine Martin Fellow at St Vincent’s Institute, and is now a Vice Chancellors Fellow at RMIT University. J.A.R. is funded by Agencia Nacional de Ciencia y Desarrollo (Chile), FONDECYT 11181000 and FONDAP 15130011. This work was supported by the Medical Research Council Core funding to the MRC LMCB (MC_U12266B). D.J.H. was supported by the British Heart Foundation (CS/14/3/31002), the National Institute for Health Research University College London Hospitals Biomedical Research Centre, Duke-National University Singapore Medical School, Singapore Ministry of Health’s National Medical Research Council under its Clinician Scientist-Senior Investigator scheme (NMRC/CSA-SI/0011/2017) and Collaborative Centre Grant scheme (NMRC/CGAug16C006), and the Singapore Ministry of Education Academic Research Fund Tier 2 (MOE2016-T2-2-021). This article is based upon work from COST Action EU-CARDIOPROTECTION CA16225 supported by COST (European Cooperation in Science and Technology).

## Notes

Translational perspective Hydralazine is used clinically as a treatment for patients with hypertension and chronic HF, and experimental studies have known it to have antioxidant and anti-apoptotic effects. In our study, we show that administration of hydralazine immediately prior to reperfusion inhibited IRI-induced mitochondrial fission, and reduced MI size. These findings raise the possibility of repurposing hydralazine as a novel potential cardioprotective therapy, which can be administered to AMI patients immediately prior to reperfusion by primary percutaneous coronary intervention, to reduce MI size and prevent HF.
